# Makerere University-Uganda Heart Institute collaborative cardiovascular disease training and research since 1988

**DOI:** 10.4314/ahs.v22i2.11S

**Published:** 2022-08

**Authors:** Emmy Okello, Lameck Ssemogerere, Isaac Ssinabulya, James Kayima, Damalie Nakanjako, Elias Sebatta, Sulaiman Lubega, Michael Oketcho, Tom Mwambu, Peter Lwabi, Cephas Mijumbi, John Omagino

**Affiliations:** 1 Uganda Health Institute, Kampala, Uganda; 2 School of Medicine, Makerere University College of Health Sciences

**Keywords:** Cardiovascular research, Uganda Heart Institute, Makerere University College of Health Sciences, Cardiology training

## Abstract

**Background:**

Makerere University College of Health Sciences has been collaborating with the Uganda Heart Institute to build capacity for research, training and clinical care in cardiovascular medicine for the last 34 years to appropriately respond to rising societal needs for advanced cardiovascular care which was lacking before this period.

**Aim:**

To describe the major milestones in the MakCHS-UHI cardiovascular training collaboration and chart way for future collaborations.

**Method:**

This short communication highlights some of the salient features and important milestones in the collaboration journey of the two institutions.

**Conclusion:**

Clinical centres of excellence in specialised fields of health care, such as the Uganda Heart Institute for Cardiology, provide a conducive academic environment for MakCHS clinical scientists to provide high quality evidence-based care to meet societal needs.

## Makerere-UHI training collaboration for 34 years

The Uganda Heart Institute (UHI) was established jointly by Makerere University, the Uganda Heart Foundation, Mulago Hospital and Ministry of health over 30 years ago to provide cardiovascular care, training and research. Right from its inception, the UHI has been collaborating with Makerere University through training by providing a teaching site for Makerere University undergraduate and postgraduate students. Most UHI faculty trained at Makerere University medical school, now called Makerere University College of Health Sciences (MakCHS), and are trainers for programs at MakCHS as clinicians, supervisors of graduate research and investigators on collaborative research projects. Makerere University faculty with super-specialised training in cardiovascular sciences participate in training of fellows at the UHI.

The UHI has a 10-year-old cardiovascular sciences training program where doctors completing their masters level training in internal medicine, paediatrics, surgery, anaesthesia and critical care are invited to join a three-year fellowship in adult cardiology, paediatric cardiology, adult cardiac surgery, paediatric cardiac surgery or cardiac anaesthesia and critical care. Two fellows per program are competitively selected annually resulting in eight fellows/year and 24 at any one time.

The UHI has seven main clinical departments of adult cardiology, paediatric cardiology, adult cardiac surgery, paediatric cardiac surgery, cardiac anaesthesia and critical care and cardiac nursing. These departments are supported by an accredited clinical laboratory, pharmacy, radiology and a nutrition and cardiac prevention unit. As Uganda's only tertiary center for cardiovascular disease, UHI sees >20,000 outpatients and admits >5000 inpatients each year and performs >15,000 echocardiograms and >20,000 electrocardiograms annually. UHI also has one of the only functional public cardiac catheterization laboratories in Uganda, performing over 1000 diagnostic and therapeutic procedures a year, as well as a busy open-heart surgical program, performing over 500 cases each year. UHI also offers short term courses in cardiac diagnostics to doctors and physicians around the country. All courses at UHI are accredited by the Uganda Medical and Dental Practitioners Council (UMDPC). Through its different programs, the UHI has over 30 faculty involved in the daily management of patients with cardiovascular diseases as well as teaching and supervision of research. Impact of academic research at MakCHS and UHI

Physicians from Makerere University College of Health Sciences (MakCHS) and UHI have been collaborating for over a decade to conduct world-class research on cardiovascular diseases. From inception, this research partnership has included an intentional mentoring component, with early career investigators included on nearly 100% of publications^1^, ^2^. Several joint training grants as well as individual mentoring of interested medical students, residents, and fellows have successfully launched careers in CVD-related research and inspired further research training.

Makerere University has hosted several research training grants over the last 15 years which has resulted in further development of UHI faculty. The most recent grant has been the Training Health Researchers into Vocational Excellence (THRiVE) consortium which was established in 2009 as a regional network of research excellence partnering with some of the best universities and research institutes in East Africa together with Cambridge University and London School of Hygiene and Tropical Medicine both in UK. THRiVE resulted in over 450 publications and 57 masters research fellows, 31 PhDs, 19 postdocs (including UHI faculty).

NURTURE was the other prominent grant that supported development of UHI faculty. NURTURE aimed to develop a sustainable culture for supporting research training and mentoring for career development of upcoming faculty fostering continuous professional development and retention of independent researchers. In total, 70 junior and mid-level faculty at MakCHS and affiliated institutions were supported to carry out research in various non-communicable diseases (NCD) including Cardiovascular-diseases (CVD). Many junior and senior level UHI faculty were supported through this grant.^3^, ^4^

Through these collaborative capacity building grants, the UHI and indeed Uganda has become a world leader in rheumatic heart disease (RHD) research, providing much needed data and evidence on RHD epidemiology, pathogenesis and new treatment approaches. This knowledge has resulted in improved diagnostic capacity of the health care workers and better outcomes of patients with RHD over time.^5^, ^6^ A key outcome of our RHD work has been the established of the Uganda National RHD registry which has become a platform for major studies that have influenced policy in Uganda and around the world.

Other training partnerships between MakCHS and UHI have included the NIH funded Medical Education Partnership Initiative (MEPI) CVD linked award. Senior faculty at the UHI and several mid-level faculty were beneficiaries of this training collaboration for their doctoral and masters level studies^7^. The MEPI-CVD linked award supported a total of 25 masters' students, 7 non degree fellowship training and 3 PhD. This collaboration also resulted in training the first two adult interventional cardiologists in Uganda with significant growth in cardiovascular capacity in Uganda^8^.

## Conclusion

Clinical centres of excellence in specialised fields of health care, such as the Uganda Heart Institute for Cardiovascular medicine, provide a conducive academic environment for MakCHS clinical scientists to provide high quality evidence-based care to meet societal needs.

## The future

The MakCHS-UHI collaboration will continue to build capacity in cardiovascular medicine while providing a platform that can be benchmarked by other yet to be developed specialty areas.

## Figures and Tables

**Figure 1:**
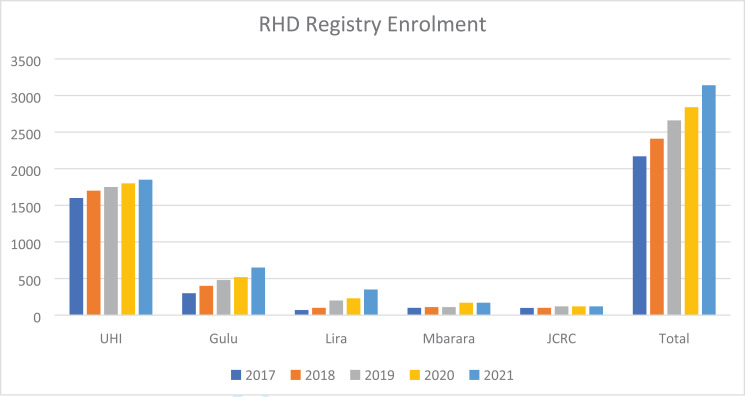
Graph showing the Uganda RHD Registry enrolment over the last 5 years. (Source: UHI)
